# 

**Published:** 1889-11-02

**Authors:** 


[Registered at the General Post Office as a Newspaper for transmission in the United Kingdom and Abroad.

Per Annum, 6s. 6d., post free.
Monthly Parts, 6d.; post free 7½d.
Ditto per Annum, 7s. 6d., post free.

### THE HOSPITAL

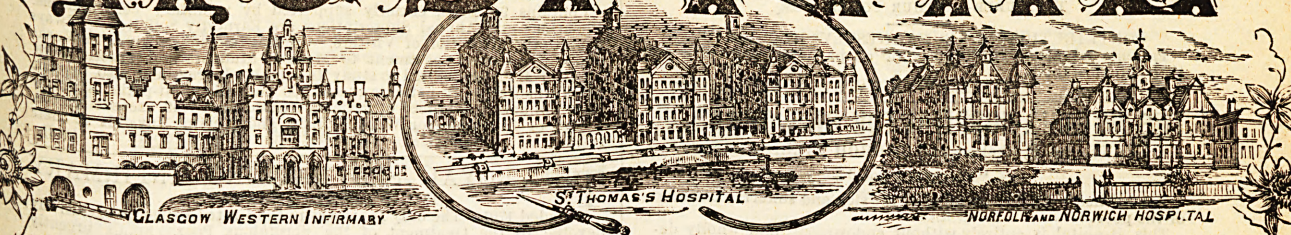

A WEEKLY INSTITUTIONAL JOURNAL OF

**Science, Medicine, Nursing & Philanthropy.**

SATURDAY, NOVEMBER 2nd, 1889.

**The Medication of the Mind** - - - - - - - 65
**Words of Consolation.**—Long Suffering - - - - 66
**The Doctor's Pulpit.**—Professional Pressure - - - 67
**Antiseptic Ventilation for Hospitals and Sanatoriums.** By S. M. Burroughs - - - - - 68
**Hypnotism or Mesmerism.** By J. A. Manton, M.R.C.S. 69
**Modern Socialism.** By a Student of Present-Day Movements - - - - - - - - - - - 70
**Everybody's Page** - - - - - - - - - - 71
**Notes and News** - - - - - - - - - - 72
**Annotations.**—"Foreign Medical Literature"—Openings for Young Men—Londoners and their Railways - - - 74
**The Practitioner's Outlook and Retrospect:**
Medicine—Bleeding—Hammer Nose—Microbes - - 75
Surgery—Scrofula - - - - - - - - - 76
Theraphutics—Cases Treated by Ice—Locomotor Ataxy - 76
New Drugs, Appliances, and Things Medical—Revolutions in Tea—Santha - - - - - - - 77
**Cooks and Cooking** - - - - - - - - - 78
**The Hospital Workshop.** XIII.—The Jaffray Suburban Hospital, Birmingham - - - - - - - 79
**Editor's Letter-Box.**—The Electro-Magnet in Ophthalmic Surgery—Heredity—The Clapham Maternity Hospital - 80
**Scraps and Gleanings** - - - - - - - - - 80

**EXTRA SUPPLEMENT—THE NURSING MIRROR.**

**En Passant.**—King's Norton—Burton-on-Trent Infirmary—Glasgow Institutions—Nursing Lectures—Short Items—Ipswich Nurses' Home—Notes from University College—Wigan Infirmary Nurses - - - - - - - xxi

**Cottage Hospital Matrons.** III.—Household and Social Duties - - - - - - - - - - - xxiii

**The Two Surgeons** (concluded) - - - - - - xxii

**Everybody's Opinion.**—Village Nurses—Ladies as District Nurses - - - - - - - - - - xxiii

**Appointments** - - - - - - - - - - xxiv

**Vacancies** - - - - - - - - - - - xxiv

**Notes and Queries—Amusements** - - - - - - xxiv

